# Chitosan in Modern Pharmacotherapy: Chitosan Nanoparticles and Delivery Systems

**DOI:** 10.3390/pharmaceutics18091118

**Published:** 2026-09-06

**Authors:** Lolita Kuršvietienė, Robertas Lažauskas, Inga Stanevičienė

**Affiliations:** 1 Department of Biochemistry, Medical Academy, Lithuanian University of Health Sciences, A. Mickeviciaus St. 9, LT-44307 Kaunas, Lithuania; 2 Institute of Physiology and Pharmacology, Medical Academy, Lithuanian University of Health Sciences, LT-50161 Kaunas, Lithuania

**Keywords:** chitosan, chitosan-based systems, chitosan nanoparticles, drug delivery, therapy

## Abstract

The development of effective delivery systems remains an important challenge in modern pharmaceutical and biomedical research, driving the development of biocompatible materials with versatile functional properties. This review provides an integrated overview of chitosan-based delivery systems, focusing on chitosan nanoparticles, chitosan derivatives, and hybrid systems. Particular emphasis is placed on the relationship between the structural and functional characteristics of chitosan-based systems and their potential applications in pharmaceutical and biomedical fields. In addition to established applications, emerging approaches involving nucleic acid delivery, gene therapy, theranostics, and other biomedical applications are discussed. A further focus of this review is the consideration of critical quality attributes relevant to the development of GMP-oriented chitosan-based systems, providing a perspective that links material characteristics with quality and translational requirements. By integrating these aspects, this review highlights the versatility of chitosan as a platform for the development of innovative pharmaceutical and biomedical delivery technologies.

## 1. Introduction

Chitosan is a natural polysaccharide obtained by partial deacetylation of chitin and is highly valued for its biocompatibility, biodegradability and versatility for chemical modifications [1,2]. Its positively charged amino groups give it unique properties, including mucoadhesion, the ability to open epithelial tight junctions and strong interactions with negatively charged biological membranes, which help to increase the bioavailability of drugs, especially biotechnologically derived therapeutic molecules [3]. The number of scientific publications on chitosan has increased significantly, now reaching several thousand articles per year [4]. Chitosan in particular has received exceptional attention as a multifunctional biopolymer in the pharmaceutical field due to the advantages of chitosan nanoparticles (ChNPs): they help to improve the pharmacokinetics of active molecules (extending their circulation time in the blood and reducing unwanted clearance), their pharmacodynamics (increasing the concentration of the drug in the target tissue) and drug solubility [4]. Beyond its function as a drug delivery carrier, chitosan exhibits antimicrobial, anti-inflammatory, antioxidant, and tissue-regenerative activities, making it a promising material for diverse biomedical applications [5,6]. Since 2015, researchers have increasingly focused on chitosan derivatives to improve their solubility, stability, and targeted bioefficacy [4,7]. Recently, significant progress has been made in the field of chitosan-based nanoparticles acting as nanocarriers, which are applied in drug encapsulation, gene therapy, theranostics, oncology and inflammatory diseases, as well as vaccinology [8,9]. Given this rapid growth, the aim of this review is to systematically present the scientific achievements of the last decade related to the use of chitosan in pharmacotherapy, discussing its pharmacological properties, various delivery systems, targeting strategies, and potential in gene therapy.

*Scope and Literature Search strategy*. PubMed and ScienceDirect were searched for English-language records published between January 2015 and June 2026, with earlier foundational works incorporated through reference list screening. Searches were run independently by three authors. Records were first screened by title, abstract and keywords; those retained at this stage were then read in full and included or excluded according to the eligibility criteria. Studies were included if they reported the development, characterization, or evaluation of chitosan-based drug encapsulation or targeted drug delivery systems and assessed at least one pharmaceutical outcome such as drug loading, release kinetics, targeting efficiency, bioavailability, therapeutic efficacy, or biocompatibility. Studies focusing exclusively on chitosan synthesis, extraction, non-pharmaceutical applications, conference abstracts, editorials, and non-English publications were excluded. The final set of records was analyzed and the evidence synthesized.

## 2. Pharmacological Properties of Chitosan

Chitosan is a natural poly-(1→4)-2-amino-2-deoxy-β-D-glucose polymer derived from the partial deacetylation of chitin, a polysaccharide found in the cells of crustaceans, insects, and fungi [2]. This biopolymer is insoluble in neutral solutions but dissolves in certain organic acids (e.g., acetic, lactic) and inorganic acids (e.g., hydrochloric acid), where its protonated amino groups acquire a positive charge [2]. This protonation in an acidic environment gives chitosan a polycationic character. The positively charged chains interact strongly with negatively charged components of cell membranes and mucosa, leading to its exceptional mucoadhesive properties [3]. This property, combined with its ability to temporarily open intercellular tight junctions in the epithelium, leads to increased drug permeation across the mucosal barrier, which is particularly beneficial for large-molecule drugs like peptides [10,11,12,13].

The control over chitosan’s solubility and other physicochemical parameters depends on its structural features, including its crystalline structure, degree of deacetylation, molecular weight (MW), and the distribution of acetyl groups along the chain [2]. Modulating these parameters through chemical modification can significantly alter chitosan’s properties, allowing it to be tailored for specific pharmacotherapeutic needs [4,7]. Moreover, the ability of chitosan to form polyelectrolyte complexes with nucleic acids enables the stable encapsulation of siRNA, mRNA, and plasmid DNA into polyplexes, protecting them from nuclease degradation and thereby facilitating gene delivery applications [14,15].

Recent research shows that chitosan not only functions as an auxiliary carrier but also exhibits various pharmacological activities. It is biocompatible, biodegradable, non-toxic, and has very low allergenicity [2]. A broad spectrum of its biological effects has been observed: chitosan inhibits the growth of numerous microorganisms, providing an antimicrobial and antifungal effect by damaging microbial membranes, causing changes in permeability and loss of cellular content [11,16]. It also possesses antioxidant properties, promotes tissue regeneration (used in wound healing), and can have antineoplastic and even anti-diabetic effects [6]. Because of these properties, chitosan is considered a versatile biopolymer in biomedicine. Its biocompatibility, biodegradability, ability to chelate metal ions, and environmental stability are particularly emphasized [2]. These characteristics, combined with its pH-sensitivity and the possibility of introducing new functional groups into the polymer chain, have led to a surge of interest in chitosan derivatives for developing innovative drug carriers in recent years [4,7]. Chemical modifications enhance solubility, mucosal adhesion, biocompatibility, pH sensitivity and add new beneficial properties [17].

**Figure 1:**
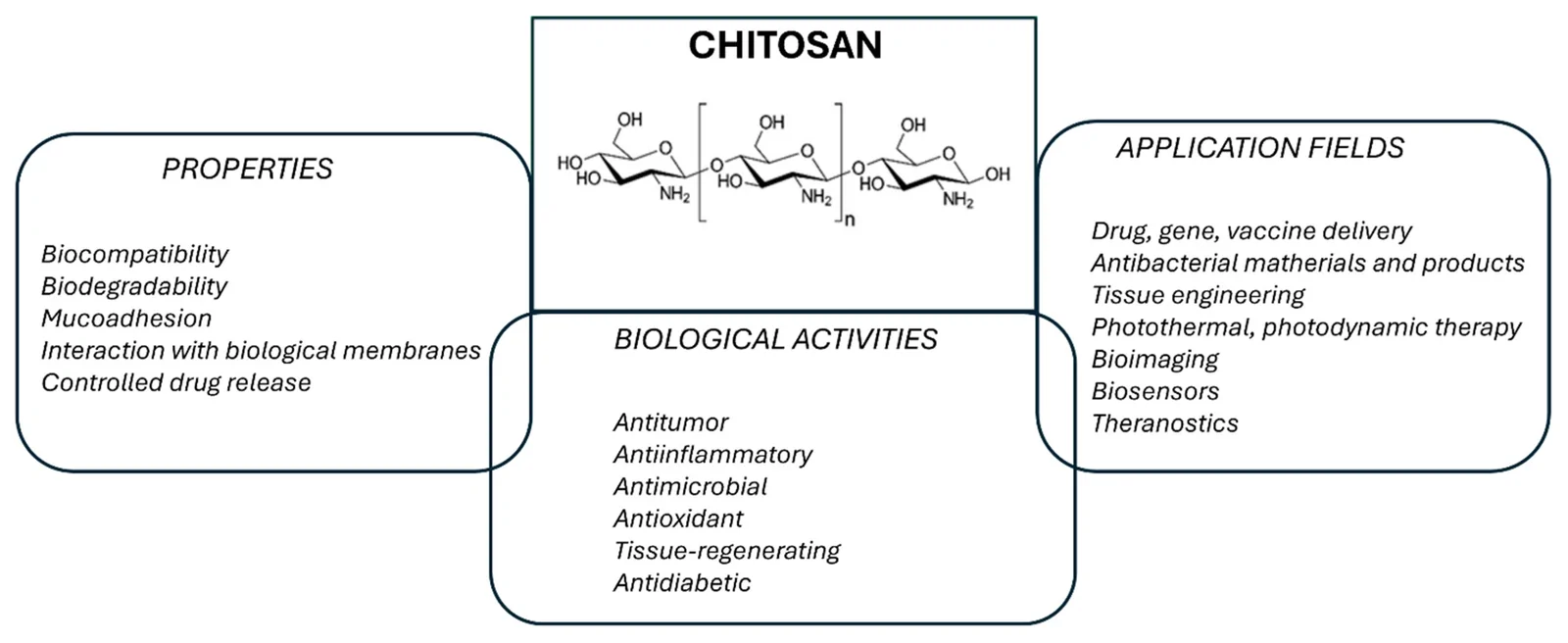
The structure, properties, biological activities and application fields of chitosan.

Main properties, biological activities and application fields of chitosan are given in Figure 1.

## 3. Chitosan-Based Delivery Systems

The chemical structure of chitosan (a linear β-(1→4)-glucosamine chain) allows for the formation of diverse drug delivery systems. Due to its polymeric nature and the abundance of reactive amino groups, chitosan can be cross-linked with a variety of reagents and polymers to form carriers of different architectures, including nanoparticles, microparticles, hydrogels, nanogels, fibers, and films [18]. Among these, particulate carriers constitute the primary focus of this section and are commonly classified according to their size and morphology. Based on size, they are generally divided into nanoparticles and microparticles. According to IUPAC terminology, nanoparticles are particles with at least one dimension between 1 and 100 nm (in some cases extending up to 500 nm), while microparticles are characterized by dimensions in the micrometer range [19]. Furthermore, particles ranging from 100 to 1000 nm, which fall between nanoparticles and microparticles in size, can be classified as submicron particles.

Particulate chitosan-based nano- and microcarriers are extensively used for drug encapsulation, as they enhance drug bioavailability while enabling controlled and targeted release. Encapsulation within chitosan-based systems improves drug stability, reduces systemic toxicity, enhances cellular uptake, and protects therapeutic agents from premature degradation. A wide range of therapeutics, including antimicrobial, antitumor, anti-inflammatory, and analgesic agents, have been successfully incorporated into chitosan-based carriers [20]. In particular, ChNPs are highly effective for the encapsulation of hydrophobic compounds, thereby improving their aqueous solubility and stability [14].

Nanospheres are solid, spherical polymeric nanoparticles in which the drug is homogeneously distributed throughout a dense polymer matrix via entrapment, dissolution, or surface adsorption [21,22]. In contrast, nanocapsules consist of a core–shell structure, featuring a liquid (aqueous or oily) core surrounded by a polymeric shell, where drugs are typically confined within the core or, in some cases, attached to the surface [23]. Drug release from nanospheres is primarily governed by diffusion and/or polymer matrix degradation, enabling sustained and controlled release profiles. In nanocapsules, drug release is mainly controlled by diffusion through the polymeric membrane and may also exhibit an initial burst release upon exposure to the biological environment [24]. Therefore, the selection of an appropriate chitosan-based nanoparticulate system depends on the physicochemical properties of the drug, the biological environment of the target site, and the required circulation stability and clearance behavior [22,25].

Among these delivery systems, hydrogels and nanogels have attracted considerable attention owing to their high water content, biocompatibility, biodegradability, and tunable physicochemical properties. Chitosan hydrogels can be prepared by either physical or chemical cross-linking, resulting in networks stabilized by non-covalent or covalent interactions, respectively [26]. Their swelling behavior and drug release profiles can be tailored by modifying the cross-linking strategy, allowing the development of stimuli-responsive systems, particularly pH-sensitive hydrogels for site-specific drug delivery. For example, they remain stable in the acidic environment of the stomach but swell and release the drug in the neutral or alkaline environment of the intestine. Such systems are particularly useful in colonic therapy for treating inflammatory bowel diseases and colorectal cancer [11,27].

Chitosan nanogels combine the advantages of hydrogels with nanoscale dimensions, providing protection of encapsulated therapeutic agents, improved circulation stability, and controlled drug release in response to physiological stimuli [28]. For example, they create a hydrated environment that effectively protects encapsulated proteins and peptides from enzymatic degradation [14].

The development of chitosan nanodrugs considers numerous design parameters—including particle size, shape, surface charge, and degree of cross-linking—as these factors determine the drug release rate and distribution within the body [7]. Research indicates that by optimizing these parameters (e.g., using techniques like ionic gelation, emulsification, and coacervation), it is possible to encapsulate both hydrophilic and hydrophobic compounds, protecting them from premature degradation and ensuring controlled release at the desired location [4,7,16,29]. Therefore, chitosan delivery systems are versatile and can be applied to transport a wide variety of active substance classes.

### 3.1. Main Types of Chitosan Nanoparticles

Based on the composition and properties, nanoparticles are divided into several categories: inorganic, organic, polymeric, and hybrid nanomaterials [30]. Chitosan-lipid and chitosan-polymer nanoparticles are advanced hybrid drug-delivery systems that pair chitosan’s natural biodegradability with the structural advantages of lipids or other polymers; they are designed to enhance drug stability, bioavailability, and controlled release across biological barriers. The most recent and innovative materials are hybrid nanocomposites combining inorganic and polymeric components. Specific properties and fields of biomedical application of different types of chitosan nanoparticles and pure chitosan nanoparticles are given in Table 1.

In addition to nanoparticle composition, the fabrication method plays a crucial role in determining the properties and performance of chitosan nanoparticles. Numerous fabrication methods have been developed for the preparation of ChNPs, including ionotropic gelation, polyelectrolyte complexation, emulsification and cross-linking, reverse micelle formation, microemulsion, solvent evaporation, precipitation, spray drying, supercritical CO_2_ processing, self-assembly, milling, high-pressure homogenization, and ultrasonication [35]. Among these, selected representative techniques are briefly described to illustrate different strategies for the preparation of chitosan-based drug delivery systems. Ionotropic gelation and polyelectrolyte complexation are most commonly used because these techniques are simple and do not require high shear pressures or organic solvents [55]. Ionotropic gelation is based on electrostatic interaction between cationic chitosan and an anionic cross-linker, such as tripolyphosphate (TPP), sulphate, citrate or alginate. This method is simple, aqueous-based, cost-effective, and scalable. This cross-linking process results in stable particles, whose size varies in the range of 50–1000 nm. Such nanoparticles with high drug encapsulation efficiency can be used for drug delivery, gene therapy, vaccine delivery and wound healing [66]. Another strategy is the formation of polyelectrolyte complexes with polyanions, which provides nanoparticles with stability through electrostatic interactions and controlled release [11,16]. This method is based on complexation between oppositely charged polyelectrolytes. Hyaluronic acid, heparin and dextran sulfate are used as cross-linking agents in this method. The size of particles obtained by this method is 100–800 nm. Such particles are used for protein delivery and tissue engineering. Self-assembly methods, in which chitosan is chemically modified with hydrophobic groups or mixed with phospholipids, allow for the creation of amphiphilic nanoparticles suitable for the transport of lipophilic drugs [29,67]. Microspheres obtained by spray drying offer controlled drug release, making them particularly suitable for oral administration [11,12]. In the direct covalent cross-linking method, the cross-linker (glutaraldehyde, genipin, polyethylene glycol, or monofunctional agents) reacts directly with the primary amine (–NH_2_) or hydroxyl (–OH) groups on the chitosan chain, forming permanent covalent bonds. Covalent modification is chosen when nanoparticles need to be highly stable, resistant to pH changes, and capable of long-term sustained hydrophobic drug release. Macro- and micro-properties such as permeability, wetting, mechanical properties, and chemical resistance are improved by the crosslinking process of chitosan biopolymer with, e.g., glutaraldehyde [35].

### 3.2. Chitosan Derivatives and Hybrid Systems

Numerous chemical chitosan derivatives and hybrid chitosan systems have been developed to improve the physicochemical and biological properties of native chitosan and expand its biomedical applications. Chemical chitosan derivatives are obtained by introducing functional groups into the chitosan polymer, thereby enhancing properties such as solubility, mucoadhesion, stability, cellular uptake, and endosomal escape. In contrast, hybrid chitosan systems are formed by combining chitosan with organic or inorganic materials to integrate complementary physicochemical and biological properties into multifunctional nanocarriers. Given the extensive diversity of chitosan-based systems, this section focuses on representative examples that have been widely investigated and have demonstrated significant potential for drug delivery applications. Their key advantages, limitations, and representative applications are summarized in Table 2.

## 4. Routes of Drug Administration

ChNPs have been studied for various routes of administration due to their mucoadhesive and permeation-enhancing properties. Because of these characteristics, chitosan is highly promising for developing non-invasive drug delivery methods.

The oral route is one of the most widely researched. The addition of chitosan to oral formulations helps protect active compounds from the acidic environment of the stomach and from enzymatic degradation, thereby improving their absorption in the small intestine [11,12,17]. For example, in an in vivo rat model, chitosan nanocapsules have been shown to significantly increase the absorption of the peptide hormone salmon calcitonin and prolong its therapeutic effect. This occurs because the polymer adheres to the intestinal epithelium and temporarily increases its permeability [79]. This property is particularly crucial for peptide drugs like insulin, which are typically degraded rapidly in stomach acid [80]. For example, using a type 1 diabetic rat model, oral administration of O-carboxymethyl chitosan/sodium alginate hydrogel loaded with insulin (OCMC/SA-Ins) controlled blood glucose levels better and showed a relatively high bioavailability in comparison with insulin injection. The OCMC/SA-Ins nanohydrogel had a high pharmacological availability level of 6.57%, which far exceeded the level of free insulin (0.3%) [81].

Intranasal (nasal) drug delivery has also advanced significantly with the use of chitosan carriers. In vivo and in vitro studies demonstrated that positively charged ChNPs adhere to the nasal mucosa and can transport drugs—including small molecules and nucleic acids—directly to the central nervous system via the olfactory region, bypassing the blood–brain barrier; the transport of drugs across the blood–brain barrier is significantly enhanced by the functionalization of ChNPs [10,13,14,82]. Animal studies have shown that chitosan-based nasal formulations prolong the residence time of a drug in the nasal cavity due to strong polymer adhesion to mucin, which increases the bioavailability of the active substance in brain tissue [10,83]. In vivo and ex vivo studies demonstrated the efficiency of chitosan nanoparticles containing a particular drug for the treatment of many neurological diseases: Alzheimer’s disease, Parkinson’s disease, Huntington’s disease, epilepsy, schizophrenia, brain tumor, ischemic stroke [36,84,85,86].

Chitosan is also applied in ophthalmology. Chitosan-enriched eye drops allow for a longer interaction between the drug and the corneal surface. This increases the bioavailability of the drug in eye tissues and ensures a longer-lasting effect [87]. Lacrimera^®^, a chitosan-N-acetylcysteine formulation evaluated in a retrospective case series, demonstrated good tolerability and effectiveness in dry eye disease, increasing tear film stability and thickness. These effects are thought to be related to the mucoadhesive properties of the polymer, which may prolong residence on the ocular surface [87]. Using an in vitro mucin interaction model and ex vivo ocular permeation studies, Silva et al. found that electrostatic interactions between chitosan/sodium tripolyphosphate (TPP)-hyaluronic acid nanoparticles and the negatively charged sialic acid residues of mucin altered the rheological behavior of the formulation and increased the precorneal residence time of ceftazidime, an antibiotic used to treat ocular infections caused by *Pseudomonas aeruginosa*. The mucoadhesive properties of the nanoparticles promoted prolonged drug release and enhanced ocular permeation, potentially improving drug absorption and reducing the frequency of administration compared with conventional eye drops [88]. Recent reviews indicate that viscosity enhancers reduce drainage and extend ocular surface contact time, which is critical for therapeutic exposure. Accordingly, typical viscosity targets are in the range of ~15–25 cP and retention times of ~10–30 min, depending on formulation type and mucoadhesive properties [89].

For pulmonary delivery, inhaled ChNPs have shown success in lung cancer models. They provide a localized effect while reducing systemic toxicity [11,30]. Evidence for inhaled chitosan nanoparticles in lung cancer has been obtained mainly from A549 and SPC-A1 lung cancer cell models, Sprague-Dawley rat pulmonary deposition/pharmacokinetic studies, and mouse lung metastasis models employing inhaled chitosan-siRNA nanoparticles [90,91].

It is well known that aerodynamic particle size is a key determinant of lung deposition. Multiple recent studies confirm that particles in the range 1–5 µm achieve optimal deposition in the lower respiratory tract. Particles larger than 5 µm deposit in the upper airways, while those which are less than 1 µm may be exhaled. More specifically, particles of 2–5 µm show the highest deposition efficiency in inhalation systems, as demonstrated in recent experimental and computational studies [92].

Chitosan carriers are also successfully applied through local and parenteral routes. Transdermal delivery utilizes chitosan hydrogels and films in pharmacotherapy, for instance, in wound dressings. Here, the polymer performs multiple functions: it forms a moist, protective film, has hemostatic (blood-clotting) properties, supports tissue regeneration and protects against infections due to its antimicrobial activity [18,93]. These statements are based mainly on in vitro skin-cell studies and in vivo rodent wound-healing/burn models [93].

Parenteral (injectable) delivery of chitosan hydrogels or nanoparticles is being researched as a carrier for anti-cancer drugs. In summary, chitosan nanotechnology is considered an advanced delivery platform for various routes of administration; all drug forms (oral, nasal, ophthalmic, transdermal, injectable) can be improved using chitosan derivatives [18].

## 5. Drug Delivery by Passive, Active and Stimulus-Responsive Targeting

A key goal in modern pharmacotherapy is to ensure that a drug reaches the diseased tissue at the highest possible concentration while minimizing its effect on healthy organs. Chitosan nanocarriers contribute to this goal by enabling both passive and active drug targeting.

*Passive targeting.* Passive targeting is one of the most important mechanisms underlying the application of chitosan nanoparticles in oncology. Following intravenous administration, ChNPs may accumulate within tumor tissue through the Enhanced Permeability and Retention (EPR) effect [4]. This phenomenon arises because tumor blood vessels are often poorly developed and highly permeable, allowing nanoparticles to extravasate into the tumor microenvironment. In addition, impaired lymphatic drainage within tumors may promote prolonged retention of nanoparticles at the target site [4,94]. As a result, the EPR effect may contribute to increased drug concentrations within the tumor while minimizing systemic toxicity and reducing exposure to healthy tissues. The ability of ChNPs to exploit the EPR effect is strongly influenced by their physicochemical properties, including particle size, shape, and surface charge, which can be optimized through adjustments in formulation composition and fabrication methods [94]. However, the EPR effect is increasingly recognized as a context-dependent phenomenon, with considerable variability among tumor types and between preclinical models and human cancers [95].

*Active targeting.* Furthermore, engineering techniques allow for the active targeting of ChNPs to a specific site. By functionalizing the polymer’s surface with ligands such as folic acid, antibodies, or peptides, the particles can selectively recognize and bind to cells that express specific receptors [7]. For example, many epithelial cancer cells overexpress folate receptors (FR) on their surface. Folic acid-coated ChNPs can specifically bind to these cells and deliver cytotoxic drugs inside, increasing the effectiveness of the therapy [96,97]. Similar studies have been conducted with transferrin or antibody conjugates to achieve specific cellular uptake [67,98]. Gaspar et al. reported that FR expression remains high even during chemotherapy, so attaching folate to nanoparticles helps overcome chemotherapy resistance mechanisms and improves treatment outcomes [96]. Another significant advantage is that encapsulating hydrophobic anti-cancer drugs in ChNPs allows them to be delivered without harsh solvents or surfactants, which reduces unwanted side effects. ChNPs can “load” poorly soluble chemotherapeutic compounds into their core, improving their solubility and permeability. This enables a higher drug concentration in the tumor without the excipient-related toxicity [14]. Additionally, these nanoparticles can be designed for slow drug release, allowing the anti-cancer drug to remain in the tumor tissue for a longer period, which enhances the therapeutic effect and may reduce the frequency of dosing [14].

*Stimulus-responsive targeting.* Newly engineered stimulus-responsive ChNPs have greatly advanced co-delivery technologies. These intelligent nanocarriers are designed to release their therapeutic cargo only when exposed to specific triggers—such as elevated temperatures at inflamed sites, the acidic environment of tumor cells, particular enzymatic activities, or distinct redox conditions [99]. Therefore, temperature, pH, light, redox state, ultrasound and magnetic fields are the main external and internal stimuli that trigger controlled drug release from nanoparticles. Growing research interest in stimuli-responsive chitosan-lipid hybrid carriers underscores their potential as next-generation systems for targeted cancer treatment [100]. The tumor microenvironment, which is characterized by hypoxia, acidity, and a high concentration of reactive oxygen species, provides opportunities for stimulus-responsive chitosan carriers to selectively release active compounds [98]. In acidic tumor microenvironments, pH-sensitive chitosan releases drugs, while under redox conditions with elevated GSH levels, carriers disintegrate, ensuring intracellular drug delivery [101,102]. For example, pH-sensitive mesoporous chitosan silica carriers loaded with curcumin for glioblastoma therapy demonstrated markedly greater curcumin release under acidic conditions relative to physiological pH due to protonation of the chitosan amino groups, which causes polymer chain repulsion and swelling, thereby facilitating drug diffusion [103]. Additionally, thermo-responsive properties of nanoparticles allow phase changes at physiological temperatures, enhancing drug delivery efficiency [104]. Incorporation of enzyme-recognition motifs further enables selective enzymatic degradation, supporting controlled and targeted drug release [60]. Recently, a lot of various chitosan-based nanosystems were developed for photodynamic cancer therapy; e.g., a nanosystem containing the photosensitizer, chlorin e6 (Ce6), which is able to generate highly reactive singlet oxygen and induce DNA and mitochondrial damage in cancer cells. Moreover, the increased cytotoxic effect on cancer cells (e.g., cholangiocarcinoma cells (SNU478) was linked to better cellular uptake of nanoparticles containing Ce6 compared with free Ce6 [94].

Chitosan-based targeted delivery systems are not just being explored in oncology; they are also being applied to a wide range of diseases. Recent reviews note that chitosan formulations are being developed for treating bacterial and viral infections (e.g., for delivering antimicrobial peptides or antiviral drugs), managing inflammatory diseases (e.g., ulcerative colitis), controlling metabolic disorders (e.g., obesity, diabetes), and even for gastroesophageal reflux disease (e.g., a chitosan gel that binds stomach acid) [18,105]. Furthermore, biomaterials derived from chitosan can function as immunotherapy adjuvants (by stimulating a local immune response, for example, in vaccines) and are useful in regenerative medicine (e.g., promoting cartilage regeneration in osteoarthritis) [106,107]. Interestingly, chitosan’s antibacterial properties are even being exploited in the food industry to create antimicrobial packaging impregnated with chitosan to protect food products from pathogen growth [18]. This interdisciplinary application of chitosan highlights its potential as a versatile biopolymer platform for the therapy of various diseases.

## 6. Gene Therapy, Nucleic Acid Delivery and Theranostics

A range of polymers has been explored for use in gene delivery, falling into two main categories: natural polymers, such as chitosan (Ch), and synthetic polymers, such as poly-L-lysine (PLL), polyethyleneimine (PEI), and dendrimers. While synthetic polymers generally provide high transfection efficiency, their major drawback is the potential for in vivo toxicity, which arises from their lack of targeting ability and accumulation in cells [68]. Moreover, limitations of synthetic polymers include slow biodegradation and swift clearance from the bloodstream following parenteral administration.

In the field of gene therapy, chitosan is emerging as a promising non-viral gene delivery vector. The positive charge of the polymer backbone allows it to electrostatically complex with negatively charged DNA or RNA molecules, forming polyelectrolyte complexes (polyplexes). These chitosan-DNA complexes protect the genetic material from degradation by nucleases and facilitate its entry into cells via endocytosis [8,10]. ChNPs exhibit excellent biocompatibility and lower cytotoxicity than many classic gene carriers (e.g., pure PEI), making them widely used to transport plasmid DNA, siRNA, mRNA, and even complex CRISPR/Cas9 components into cells [8,9].

In the case of brain tumors, chitosan-based carriers have been shown to cross the blood–brain barrier and deliver doxorubicin and a small interfering RNA (siRNA) into glioblastoma cells [15]. Delivering siRNA alongside chemotherapy can suppress multidrug-resistance genes such as *MDR1* or *BCL-2*. By silencing these resistance pathways, tumor cells become more vulnerable to chemotherapeutic agents, thereby enhancing the overall cytotoxic effect [99].

Importantly, innovations over the last decade have significantly improved the transfection efficiency of chitosan vectors. For example, modifying chitosan with polycationic polymers like polyethyleneimine (PEI) has led to hybrid carriers that achieve more effective gene transfer than traditional PEI vectors alone [64]. It has also been reported that attaching specific targeting ligands to chitosan (e.g., the AS1411 aptamer, which recognizes tumor cells) can ensure highly efficient delivery of plasmid DNA into targeted cancer cells [108]. These achievements pave the way for the use of CRISPR/Cas9 gene-editing technologies. ChNPs have successfully delivered CRISPR/Cas9 plasmids into cancer cells, reducing the expression of a targeted oncogene and slowing tumor invasiveness [9]. Different targeting ligands of CRISPR-Cas9-loaded nanoparticles, such as aptamer AS1411, hyaluronic acid, lactobionic acid and folic acid, ameliorate the interaction with cancer cells due to binding to nucleolin, asialoglycoprotein and folate receptors [109]. Thus, chitosan-based gene carriers are considered one of the most advanced approaches for safe and effective gene therapy in a clinical setting.

However, effective gene therapy requires endosomal escape for the material to reach the cytoplasm. Modifications such as thiolated chitosan or chitosan conjugation with imidazole groups increase the buffering capacity, promoting the “proton sponge” effect and subsequent endosomal membrane disruption [68,110]. Comparative studies show that chitosan derivatives often have lower toxicity than synthetic polycations like PEI while providing similar transfection efficiency. A medium chitosan grafting degree of about 4.5% using low-molecular-weight PEI (1.8 kDa) has been shown to achieve high transfection efficiency while maintaining low cytotoxicity in mammalian cell models such as HEp-2, suggesting that similar low-toxicity behavior may be expected in other epithelial-derived cell lines [111].

ChNPs have rapidly evolved into multifunctional nanoplatforms capable of simultaneous therapy and diagnostics—a core principle of theranostics. Current research spans cancer imaging, gene therapy, theranostics, optical probes, and precision oncology. Chitosan-based theranostic nanoparticles achieve imaging by incorporating carbon dots, fluorescent dyes, etc. For example, nanoparticles containing RNA and carbon dots are used not only for RNA delivery to cancer cells, but also for imaging and precise diagnosis [112]. Moreover, chitosan-based biosensors made of polymers, nanoparticles, and hybrid composites enable the detection of various physiologically important compounds such as lactate, histamine, cholesterol, glucose, dopamine, hydrogen peroxide, urea, etc. Chitosan-based nanocomposites have become extensively utilized as biosensing materials across environmental, biomedical, pharmaceutical, and catalytic research fields. Recently, an emerging direction in biosensor development involves integrating liquid crystals with chitosan to create advanced nanocomposite sensing platforms [113].

## 7. Safety, Stability and Translational Challenges

The toxicological profile of chitosan as a pharmaceutical adjuvant is generally favorable. Most studies indicate that the polymer is non-toxic to mammalian cells, non-mutagenic, and exhibits low cytotoxicity at typical doses [2]. Furthermore, meticulous manufacturing processes ensure that chitosan is virtually free of protein impurities, resulting in low immunogenicity. Researchers state that its allergenic potential is minimal [2]. Nevertheless, rare cases of hypersensitivity reactions to poorly purified chitosan have been reported in patients with shellfish allergies, underscoring the critical importance of selecting and purifying the raw material to ensure safety [114].

The safety of ChNPs is influenced by their physicochemical properties, including particle size, surface charge, molecular weight, degree of deacetylation (DDA), dose, exposure duration, and route of administration [115]. Highly deacetylated and strongly cationic nanoparticles exhibit enhanced cellular uptake but may also increase membrane disruption, oxidative stress, mitochondrial dysfunction, and cytotoxicity, particularly at higher concentrations. Consequently, optimization of surface charge is essential for balancing efficacy and safety. For intravenous applications, blood compatibility is a critical consideration. Although most ChNPs formulations demonstrate low hemolytic activity, increased positive surface charge and higher doses may promote erythrocyte damage, platelet activation, and thrombogenicity. In addition, endotoxin contamination of chitosan materials can confound toxicity assessments by inducing inflammatory responses unrelated to the nanoparticles themselves [116]. Following systemic administration, ChNPs are commonly cleared by the reticuloendothelial system, leading to accumulation in the liver, spleen, lungs, and kidneys. Repeated administration may result in cumulative tissue uptake and potential long-term inflammatory or organ-specific effects, although comprehensive chronic toxicity data remain limited. Sterility and storage conditions are also important determinants of safety. Sterilization methods such as gamma irradiation, autoclaving, ethylene oxide treatment, and sterile filtration may alter nanoparticle size, zeta potential, polymer integrity, drug loading, and release characteristics. Likewise, temperature, humidity, pH, and oxygen exposure during storage can affect nanoparticle stability, aggregation, degradation, biodistribution, efficacy, and toxicity profiles.

Despite numerous successful laboratory and preclinical studies, integrating chitosan into routine clinical practice faces several challenges. The main challenges associated with chitosan as a natural biomaterial include variability in its molecular weight, degree of deacetylation, and source, which can affect solubility, viscosity, and other physicochemical properties, thereby necessitating standardized production methods for clinical translation [15]. First, there are difficulties with production standardization and reproducibility. Natural chitosan, depending on its source (e.g., shrimp, crabs), can have varying degrees of deacetylation, molecular weight, and impurities. This leads to batch-to-batch variability in the polymer’s properties, which complicates scaling up from laboratory synthesis to industrial production. It is difficult to ensure consistent safety and efficacy of nanoparticles across different batches [117]. Scientists propose using Quality-by-Design (QbD) principles and rigorous characterization (specifying deacetylation, MW and polydispersity) to ensure these parameters are controlled and reproducible [117]. Main Critical Quality Attributes of GMP (Good Manufacturing Practice)-oriented chitosan systems are given in Table 3.

Second, regulatory barriers pose a significant hurdle. Chitosan-based nanocarriers do not have a long history of use as approved drug products, which requires extensive preclinical and clinical data on their pharmacokinetics, toxicology, and long-term effects [131]. Both the U.S. FDA and the European Medicines Agency (EMA) emphasize the need for precise definitions of the polymeric carrier’s composition and properties. This is complex for natural polymers where batch-to-batch variations are possible [117,131]. Currently, few chitosan-containing medical products are registered (most are medical devices, such as hemostatic wound dressings), and complex chitosan nanodrugs are still in the clinical evaluation stage. The main barriers remain scalability, stability, and regulatory compliance [131].

Clinical investigations of novel chitosan formulations began in 2015. A total of fifty clinical trials have been registered to explore novel chitosan applications during 2015–2025 [132]. ChNPs represent the most widely investigated novel drug delivery systems, accounting for 61% of studies (n = 31). Chitosan scaffolds follow at 17% (n = 9), while stimuli-responsive (smart) systems comprise 12% (n = 6). Stem cell therapy contributes 6% (n = 2), whereas 3D printing and artificial intelligence each constitute 2% of the studies (n = 1 each). With respect to ChNPs applications, dental and periodontal disorders were the most frequently studied area, accounting for 81% of the studies (n = 25). Infectious diseases represented 7% (n = 2), while dermatological/wound healing, orthopedic, pulmonary, and neurological applications each comprised 3% (n = 1). Among clinical trials investigating chitosan scaffolds, the dental/orthodontic field also predominates (n = 4), followed by musculoskeletal/orthopedic (n = 2), dermatological (n = 2), and neurological (n = 1) fields. Among clinical trials investigating stimuli-responsive systems, orthopedic applications predominate (n = 3), with one study each conducted in the fields of neurology, oncology and ophthalmology [132].

Summarizing clinical investigations, it can be stated that a relatively small proportion of studies have published results, whereas the majority are either in early development stages or lack phase designation. More than 60% of studies are still in the early stages of clinical development (Phase I/II), involving relatively small participant populations. Only a limited number of studies have progressed to Phase III or IV investigations [132].

Despite the existing challenges, ongoing scientific efforts and technological progress in production methods are likely to facilitate their resolution, paving the way for broader clinical application of chitosan-based drug delivery systems. Overcoming these limitations requires standardized characterization methods, unified regulatory guidelines, and comprehensive clinical trials [133].

## 8. Conclusions and Future Directions

Recent studies have clearly demonstrated that chitosan is one of the most promising biopolymers in modern pharmacotherapy. Unique chemical and biological properties of chitosan allow this polymer to be used across various routes of drug administration, ranging from oral formulations to targeted gene therapy delivery. Current research has shown that ChNPs can improve drug pharmacokinetics, increase the concentration of active compounds in target tissues, and reduce systemic toxicity [134].

On the other hand, while chitosan-based carriers have broad applicability, their translation into clinical practice remains limited. The major hurdles are related to standardizing polymer production, eliminating batch-to-batch variability, and providing evidence of long-term safety. Moreover, the clinical advancement of ChNPs is hindered by major challenges, particularly their limited hemocompatibility and the risk of unintended off-target distribution [121,131]. Nevertheless, advancements in technology, new chemical modification methods, and an increasing number of clinical trials suggest that in the coming years, chitosan will become an increasingly important platform not only for drug delivery but also in broader biomedical fields, from tissue engineering to immunotherapy [18].

In conclusion, over the past decade, chitosan has evolved from a polymer that was primarily an excipient into one of the most significant subjects of scientific research in pharmacotherapy. Its clinical potential is immense, and the remaining challenges open the door for further innovation.

## Figures and Tables

**Table 1:** Specific properties and integrated biomedical applications of pure chitosan nanoparticles and different types of hybrid chitosan nanoparticles.

Chitosan Nanoparticles Type	Specific Properties	Integrated Biomedical Application (Anticancer/Antimicrobial/Neurodegenerative)
Pure chitosan NPs	Strong mucoadhesion Cationic surface charge Transient tight junction modulation Enhanced epithelial permeability pH-responsive swelling Electrostatic stabilization, sensitive to ionic strength Biodegradable and biocompatible	Enhance local drug retention at the mucosal surface due to strong mucoadhesion and prolonged residence time; improve epithelial absorption mainly via paracellular transport associated with transient tight junction modulation and increased epithelial permeability; function as biocompatible carriers enhancing tumor-targeted delivery and intracellular uptake of chemotherapeutic agents; exhibit mild intrinsic antimicrobial activity through electrostatic interactions with microbial membranes; facilitate intranasal drug delivery and may contribute to nose-to-brain transport relevant for neurodegenerative applications [31,32,33,34,35,36,37,38,39,40,41].
Chitosan–lipid NPs	Enhanced permeability and cellular uptake Controlled/sustained drug release Improved solubilization of lipophilic drugs Lipid–polymer synergistic interaction Colloidal stabilization through lipid coating Biodegradable (polymer–lipid matrix)	Promote efficient cellular uptake through lipid–polymer synergistic interactions, where electrostatic attraction of chitosan to the cell surface is combined with lipid-mediated membrane interactions facilitating endocytic internalization; enhance intracellular delivery of poorly soluble therapeutic agents due to improved solubilization within the lipid phase; structural stability supports prolonged drug retention and sustained intracellular accumulation; enhance anticancer efficacy by improving chemotherapeutic drug delivery to tumor cells; exhibit enhanced antimicrobial effects via synergistic chitosan-mediated electrostatic interactions and lipid-assisted membrane interactions; improve brain delivery of lipophilic drugs via intranasal administration supported by enhanced epithelial permeability and mucosal interactions [42,43,44].
Chitosan–polymer NPs (PLGA, PEG NPs)	Controlled and sustained drug release PEGylation-induced reduced opsonization (immune evasion) Structural and colloidal stability due to polymer core and surface shielding Tunable matrix properties Biodegradable (polymer-dependent degradation)	Enable prolonged systemic circulation of NPs through reduced opsonization and enhanced structural stability; enhance anticancer efficacy via controlled and sustained drug release, leading to increased drug accumulation in tumor and reduced systemic clearance; support antimicrobial therapy by maintaining long-term bioactive drug concentrations and improving delivery efficiency; facilitate sustained delivery to the central nervous system for neurodegenerative disease applications through controlled release and polymer-mediated protection and transport [36,45,46,47].
Functionalized chitosan NPs (HA-, CPP-NPs)	Receptor-mediated targeting (HA–CD44) Cell-penetrating peptides (CPP) mediated membrane translocation Enhanced cellular uptake Intracellular trafficking and endosomal transport Surface functionalization-dependent interactions Biodegradable chitosan-based core	Enable receptor-mediated targeting via hyaluronic acid (HA) interaction with CD44 receptors and peptide-assisted membrane translocation; enhance selective anticancer efficacy by promoting delivery of therapeutic agents into tumor cells and their intracellular accumulation; support antimicrobial activity through surface functionalization-dependent interactions with microbial membranes; facilitate intracellular transport processes relevant for neurodegenerative disease applications [40,48,49,50,51,52].
Chitosan–metal/inorganic NPs	Chitosan coating–mediated core stabilization and reduced metal cytotoxicity ROS generation and modulation Plasmonic/optical properties (Au, Ag systems) Multifunctional surface properties	Stabilize inorganic nanoparticle cores and reduce metal cytotoxicity via chitosan coating; preserve plasmonic/optical properties (Au, Ag systems) and modulate ROS activity; enable ROS-mediated and photothermal anticancer effects with imaging capability (theranostic applications); exhibit strong antimicrobial activity, particularly in Ag-based systems, through ROS generation and membrane damage; are applied in neurodegenerative imaging and experimental theranostics [37,53,54,55,56,57].
Hybrid/multi-component chitosan NPs	Hierarchical multi-material architecture with synergistic physicochemical functionality Stimuli-responsive behavior Co-delivery capability for small molecules, nucleic acids, and imaging agents enabling theranostic applications	Enable hierarchical multi-material architectures with synergistic physicochemical properties, stimuli-responsive behavior, and multifunctional delivery capability, allowing co-delivery of small molecules, nucleic acids, and imaging agents; provide multimodal effects through targeted delivery, controlled release, and genetic modulation strategies (siRNA- and CRISPR/Cas-based approaches) applicable to both cancer and neurodegenerative diseases; may exhibit synergistic antimicrobial activity depending on composition; support theranostic imaging and integrated therapeutic strategies [35,40,58,59,60,61,62,63,64,65].

**Table 2:** Examples of chitosan derivatives and hybrid systems. Main advantages, disadvantages and application fields.

Chitosan Derivatives and Hybrid Systems	Advantages	Disadvantages	Application	Ref.
Trimethyl Chitosan (TMC)	Soluble across a wide pH range Higher positive charge that enhances cellular uptake Increased stability under physiological conditions High transfection efficiency	Variable degree of quaternization	Drug delivery; DNA delivery; Tissue repair; diagnostic application	[18,68]
Thiolated chitosan	Improved stability Enhanced mucoadhesive properties Favorable drug entrapment High cellular uptake Prolonged release properties	Complexity of synthesis; oxidation sensitivity	Cancer treatment; wound treatment; Tissue engineering; theranostics	[18,68]
Histidine-modified chitosan	Enhanced endosomal escape Improved transfection efficiency Improved solubility at physiological pH	Reduced overall cationic charge; complex synthesis	Gene delivery and transfection; Controlled drug delivery	[69]
Imidazole chitosan derivatives	Enhanced antifungal, antibacterial properties in comparison with chitosan	Complex synthesis and cost	Engineering medical devices/materials	[29,70,71]
PEGylated chitosan	Improved stability and solubility Prolonged circulation half-life Reduced surface charge	PEGylation can hinder cellular uptake and endosomal escape	Targeted cancer treatment	[68,72]
Chitosan-cyclodextrin hybrids	Inclusion ability Sustained release	Weaker mucoadhesion in comparison with chitosan	Nose-to-brain drug delivery; Delivery of drugs such as nonsteroidal anti-inflammatory drugs, antimicrobial and antitumoral agents, macromolecules, or genetic materials	[73,74]
Chitosan-Silica hybrids	Enhanced mechanical, biological, and degradation properties	Poor solubility and pH limitations; slow degradation	3D cell culture, tissue engineering, protein/drug delivery; Wound healing	[75]
Chitosan–Graphene Oxide (GO) Nanocomposites	GO enhances chitosan’s mechanical barrier and antimicrobial properties GO provides a large specific surface area and drug loading capacity	Low stability	Wound healing; drug and biomacromolecules delivery; Biomedical materials (biosensors); Cartilage and bone tissue engineering	[76,77,78]

**Table 3:** Main Critical Quality Attributes of GMP-oriented chitosan systems.

Critical Quality Attribute	Estimation Method/s	Reporting	Properties Affected	Critical Process Parameters	Clinical/ Translational Relevance	Ref.
Degree of deacetylation (DDA)	1H NMR; potentiometric titration	Report method, mean ± SD per lot; variability 80–95%	Solubility; mucoadhesion; biodegradation	Deacetylation temperature/time; alkali concentration	Alters drug loading, release kinetics, mucosal PK	[118,119,120,121]
Molecular weight (Mn, Mw, PDI)	SEC/GPC–MALS	Report Mn, Mw, PDI; solvent/ standard details	Viscosity; nanoparticle size; biodistribution	Depolymerization; purification; storage	Influences clearance, RES uptake, and exposure	[118,122]
Acetyl distribution pattern	Advanced NMR; enzymatic mapping	Qualitative /quantitative reporting per lot	Charge heterogeneity; binding affinity	Deacetylation uniformity	Impacts reproducibility of drug release	[123]
Counter-ion identity	Ion chromatography; ICP-MS	Must be specified (e.g., chloride, acetate, lactate) per lot	Stability; solubility; nanoparticle size and charge; osmolarity	Neutralization; washing/dialysis	Changes biodistribution, tolerability and PK	[124]
Residual protein/ impurities	BCA/Bradford; LC-MS	Below validated thresholds	Immunogenicity; safety	Raw material sourcing; purification	Reduces hypersensitivity risk Elevated levels can induce activation of the immune system, allergic reactions and hypersensitivity	[125]
Endotoxin content	LAL assay	Route-dependent limits; ≤100 EU/g	Pyrogenicity	Sterilization; filtration; bioburden control	Direct safety impact in trials Elevated levels can distort PK, biodistribution, immunogenicity, and therapeutic efficacy outcomes	[116,126,127,128,129]
Particle size & distribution	Raman Spectroscopy; X-ray diffraction; DLS; SEM; TEM; AFM	Mean, PDI, morphology; stability	Uptake; biodistribution	Mixing; cross-linker ratio; pH	Tissue penetration and efficacy	[31]
Zeta potential	ELS	Report medium, pH; stability range	Mucoadhesion; colloidal stability	Ionic strength; surface chemistry	Affects aggregation and uptake	[31]
Drug loading and encapsulation	HPLC/LC-MS	% loading, EE, batch variability	Dose accuracy; release	Polymer/drug ratio; cross-link density	Determines therapeutic index	[128]
Release kinetics	Biorelevant media; modeling	Model fit + conditions	Controlled exposure	MW; DDA; cross-linking	Predicts dosing and PK	[41]
Sterility/bioburden	Compendial sterility tests	Route-specific compliance	Safety	Aseptic processing	Required for IND/CTA	[115]
Stability	ICH stability protocols	Shelf-life; degradation; aggregation	Potency; safety	Storage; lyophilization	Scalability and approval readiness	[130]

SEC, Size-Exclusion Chromatography; GPC, Gel-Permeation Chromatography; MALS, Multi-Angle Light Scattering; 1H NMR, Proton Nuclear Magnetic Resonance spectroscopy; ICP-MS, Inductively Coupled Plasma-Mass Spectrometry; LC-MS, Liquid Chromatography-Mass Spectrometry; LAL, Limulus Amebocyte Lysate assay; RES, Reticuloendothelial System; BCA, Bicinchoninic Acid assay; PDI, Polydispersity Index; PK, Pharmacokinetics; Mn, Number-Average Molecular Weight; Mw, Weight-Average Molecular Weight; DLS, Dynamic Light Scattering; SEM, Scanning Electron Microscopy; TEM, Transmission Electron Microscopy; AFM, Atomic Force Microscopy; ELS, Electrophoretic Light Scattering; HPLC-MS, High-Performance Liquid Chromatography-Mass Spectrometry; LC-MS, Liquid Chromatography-Mass Spectrometry; EE, Encapsulation efficiency; IND, Investigational New Drug; CTA, Clinical Trial Application; ICH, International Council for Harmonization of Technical Requirements for Pharmaceuticals for Human Use.

## Data Availability

No new data were created or analyzed in this study. Data sharing is not applicable to this article.
